# ApoB100 remodeling and stiffened cholesteryl ester core raise LDL aggregation in familial hypercholesterolemia patients

**DOI:** 10.1016/j.jlr.2024.100703

**Published:** 2024-11-16

**Authors:** Maria Teresa La Chica Lhoëst, Andrea Martínez, Eduardo Garcia, Jany Dandurand, Anna Polishchuk, Aleyda Benitez-Amaro, Ana Cenarro, Fernando Civeira, Amable Bernabé, David Vilades, Joan Carles Escolà-Gil, Valerie Samouillan, Vicenta Llorente-Cortes

**Affiliations:** 1 Experimental Pathology Department, Institute of Biomedical Research of Barcelona (IIBB)-Spanish National Research Council (CSIC), Barcelona, Spain; 2 Cardiovascular Area, Biomedical Research Institute Sant Pau (IIB Sant Pau), Barcelona, Spain; 3 Cardiovascular Area, Institut de Recerca de l'Hospital Santa Creu i Sant Pau, Institut d'Investigacions Biomèdiques IIB Sant Pau, Barcelona, Spain; 4 Biochemistry Department, Universitat Autònoma de Barcelona, Barcelona, Spain; 5 CIRIMAT, Université de Toulouse Paul Sabatier, Equipe PHYPOL, Toulouse, France; 6 Hospital Universitario Miguel Servet, IIS Aragón, Instituto Aragonés de Ciencias de la Salud, Universidad de Zaragoza, Zaragoza, Spain; 7 CIBER de Enfermedades Cardiovasculares CIBERCV, Institute of Health Carlos III, Madrid, Spain; 8 Institut de Ciència de Materials de Barcelona (ICMAB-CSIC), Campus UAB, Bellaterra, Spain; 9 Cardiac Imaging Unit, Department of Cardiology, Hospital de la Santa Creu i Sant Pau, IIB SANT PAU, Barcelona, Spain; 10 CIBER de Diabetes y Enfermedades Metabólicas Asociadas (CIBERDEM), Madrid, Spain

**Keywords:** familiar hypercholesterolemia, LDL aggregation, ApoB100, FTIR, DSC, secondary structures

## Abstract

Patients with familial hypercholesterolemia (FH) exhibit a significant residual cardiovascular risk. A new cardiovascular risk factor is the susceptibility of individual LDL particles to aggregation. This study examined LDL aggregation and its relationship with LDL lipid composition and biophysical properties in patients with FH compared to controls. LDL aggregation was measured as the change in particle size, assessed by dynamic light scattering, after exposure to sphingomyelinase, which breaks down sphingomyelin in the LDL phospholipid layer. Dynamic light scattering and transmission electron microscopy showed that LDL in FH patients exhibited smaller size and greater susceptibility to aggregation. Biochemical analyses revealed a higher cholesteryl ester (CE)/ApoB100 ratio in LDL from FH patients. Differential scanning calorimetry showed that LDL from FH patients had higher transition temperatures, indicating a more ordered CE core. Fourier transform infrared spectroscopy revealed fewer flexible α-helices (1658 cm⁻^1^) and more stable α-helices (1651 cm⁻^1^) in ApoB100 of LDL from FH patients. These structural changes correlated with higher CE content and increased LDL aggregation. In conclusion, a more ordered CE core in smaller LDL particles, combined with a higher proportion of stable α-helices in ApoB100, promotes LDL aggregation in FH patients. These findings suggest ApoB100 conformational structure as a new potential therapeutic targets within LDL to reduce cardiovascular risk in FH patients.

## Graphical abstract

**Figure:**
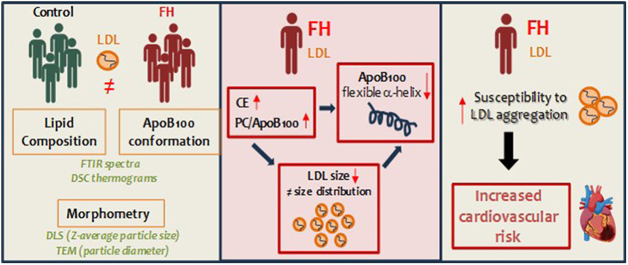

Familial hypercholesterolemia (FH) is the most common genetic metabolic disorder, affecting approximately 1 in 200–300 individuals in the general population. FH is characterized by elevated levels of LDL-C from birth. High LDL-C levels are a primary causal factor for atherosclerotic cardiovascular disease (ASCVD) and contribute significantly to overall cardiovascular risk, being closely associated with premature mortality in FH patients (1, 2). LDL-C levels are currently reduced by lipid-lowering therapies, such as statins and PCSK9 inhibitors, which have been shown to effectively decrease cardiovascular events (3, 4). Even with substantial reductions in LDL-C, significant cardiovascular risk for ASCVD and its clinical manifestations persists (5, 6, 7), attributed to the propensity of LDL to undergo modifications within the arterial intima (8). Aggregated LDL (agLDL) has been detected and isolated from atherosclerotic lesions in both experimental animal models and humans (9, 10, 11, 12). Mendelian randomization studies and randomized trials involving over 2 million participants, with more than 20 million person-years of follow-up and over 150,000 cardiovascular events, have demonstrated that exposure of the vasculature to LDL-C mechanistically causes ASCVD, and that this effect intensifies with longer durations of exposure to LDL (13). Consistently, the susceptibility of LDL to aggregation is increased in patients with coronary and peripheral atherosclerosis and is a predictor of future adverse cardiovascular events (14, 15). One of the primary cellular receptors for agLDL in human coronary vascular cells is LDL receptor-related protein 1 (LRP1) (16, 17). LRP1 is significantly upregulated by cardiovascular risk factors such as hypercholesterolemia (18), hypertension (19), and hypoxia (20) in both in vitro and in vivo models. Additionally, the soluble form of LRP1 has prognostic and predictive value in coronary artery disease (21, 22) and acute cerebrovascular events (23).

In the arterial intima, several factors play a crucial role in LDL particle aggregation. This process involves proteolytic and lipolytic digestion by local enzymes, including group V secretory phospholipase A2 (10), secretory sphingomyelinase (SMase) released by endothelial cells and macrophages (24), and mast cell chymase with chymotrypsin-like activity (25). Additionally, extracellular matrix proteoglycans trap LDL particles, contributing to LDL aggregation and foam cell formation, which is a hallmark of atherogenesis (26).

In addition to local factors within the arterial intima, diet composition plays a significant role in the individual variation for LDL aggregation susceptibility, with fat type—particularly saturated fats—being a key dietary factor (27). In contrast, increased consumption of vegetable oils and spreads enriched with plant stanols reduces LDL aggregation by altering the surface phospholipids of LDL particles (14). Oils such as camelina sativa oil, rich in alpha-linolenic acid, decrease LDL susceptibility to aggregation by modifying the balance between saturated and monounsaturated cholesteryl ester (CE) species in LDL, replacing them with polyunsaturated triglycerides (TGs) (28). According to these studies, dietary n-3 polyunsaturated fatty acids are incorporated into both the phospholipids and neutral lipids of LDL, increasing the degree of unsaturation in both the core and surface of the particles, which, in turn, influences LDL’s propensity to aggregate. Notably, LDL aggregation appears to be inversely related to specific unsaturated phosphatidylcholine species and directly correlated with unsaturated sphingomyelin (SM) species. Therefore, LDL susceptibility to aggregation varies depending on the specific phospholipid species incorporated and their degree of unsaturation. In individuals with obesity, the hepatic lipid profile influences the LDL particle lipid profile, contributing to increased susceptibility to LDL aggregation (29). Lahelma *et al.* were the first to show that the distribution of double bonds and acyl carbon numbers in TGs, SMs, and phosphatidylcholines of LDL particles closely mirrors that of the liver. Additionally, qualitative sphingolipid alterations were observed on the LDL surface and in the liver of individuals with increased susceptibility to LDL aggregation. Finally, elevated dihydroceramide and ceramide levels in the liver were associated with SM-rich LDL particles prone to aggregation. These findings underscore the significant role of dietary-induced changes in LDL lipid composition on LDL aggregation susceptibility in humans.

In addition to lipids, conformational alterations in ApoB100 lead to its degradation, a critical process in LDL aggregation (24, 30). Notably, a bidirectional and reciprocal influence between lipid and ApoB100 degradation has been reported (31, 32). Fourier transform infrared (FTIR) spectroscopy provides the exceptional advantage of capturing multi-component biochemical information from various biomolecular species, allowing for their quantification (33). This technique enables the detection of protein secondary structure alterations through an integrated and unique spectrum for each analyzed sample (34, 35). Conversely, differential scanning calorimetry (DSC) is widely used to quantify lipid levels and gain insight into their physical structure (36, 37).

Integrated lipid and ApoB100 biophysical alterations in LDLs from patients with FH have not yet been thoroughly explored. The objectives of this study are 1) to enhance understanding of lipid, morphological and structural biophysical variables in human LDL from FH patients compared to those from control subjects and 2) to investigate whether these variables influence the susceptibility of LDL to aggregation in FH patients.

## Material and Methods

### Characteristics of patients with FH and control subjects

This study includes two groups: FH patients (n = 35) and control subjects (n = 29). The FH group comprised individuals aged 23–70 years with a diagnosis of primary hypercholesterolemia, recruited from the Lipid Unit of Hospital Universitario Miguel Servet, Zaragoza, Spain. Primary hypercholesterolemia was diagnosed based on plasma off-treatment LDL-C concentrations exceeding the age and sex-specific 95th percentiles of a Spanish reference population (38). As shown in Supplementary material (supplemental Table S1), the patients included in this study exhibit a relatively low polygenic risk score, with the 90th percentile of the polygenic risk score being 1.09, indicating that this FH cohort does not consist of individuals with polygenic hypercholesterolemia. All subjects meet the clinical diagnostic criteria for FH, with a score exceeding 6 points according to the Dutch Lipid Clinic Network Core criteria. Furthermore, the presence of a pathogenic or likely pathogenic mutation in the LDLR gene ensures that our FH cohort is composed of a homogeneous group of individuals with genetically confirmed FH. Inclusion criteria included a body mass index < 30 kg/m^2^, a stable weight (±3 kg in the previous 3 months), plasma TGs < 300 mg/dl, and no intake of lipid-lowering drugs (including plant sterol/stanol supplements) in the previous 5 weeks. Exclusion criteria included alcohol consumption >30 g/day, uncontrolled type 2 diabetes (glycated hemoglobin > 8%), or any other chronic disease that could interfere with lipid metabolism. The control group included 29 subjects without previous alterations in blood lipid profile and none of them receiving hypolipidemic drugs. Clinical variables recorded during visits included diabetes, hypertension, smoking status, weight, height, waist circumference, and blood pressure. Plasma samples were obtained after overnight fasting. Total cholesterol, TGs, and HDL-C were measured using standard enzymatic colorimetric methods. LDL-C was estimated using the Friedewald formula, as all samples had TG levels <400 mg/dl. ApoB100 levels were determined using immunoturbidimetric assays adapted for the COBAS 6000/501c autoanalyzer (Roche Diagnostics, Rotkreuz, Switzerland). Patient characteristics are summarized in Table 1.

**Table 1:** Lipid profile of patients with familial hypercholesterolemia compared to control subjects

Variable	Control (n = 29)	FH (n = 35)	*P*
Sex female/male (%)	62.9/37.1	48.6/51.4	0.229
Age (years)	58.7 (17–83)	42.1 (15–71)	<0.001
Total cholesterol (mg/dl)	171.5 (103–207)	334 (251–480)	<0.001
LDL-C (mg/dl)	91.5 (40–140)	250.7 (142.9–380)	<0.001
HDL-C (mg/dl)	54.1 (31–134)	69 (50–104)	<0.001
TG (mg/dl)	96 (37–317)	98 (41–406)	0.381
ApoB100 (g/L)	0.95 (0.5–1.45)	1.84 (1.22–2.41)	<0.001

For dichotomous variables (sex), Chi-square tests were performed, while for numeric variables, Student’s *t-*tests or Mann–Whitney *U* tests were applied after assessing normality using the Kolmogorov–Smirnov test. Values are expressed as mean or median (minimum-maximum), depending on the normality of the distribution.

Chol, cholesterol; FH, familial hypercholesterolemia; TG, triglyceride.

### Ethical aspects

The study was conducted in accordance with the Declaration of Helsinki II and received approval from the Research Ethics Committee (PI23/627-SA23-30). All participants provided written informed consent prior to their involvement in the study.

### Blood collection

Blood collection and processing were conducted following the Standard Operating Procedures for Serum and Plasma Collection outlined by the Early Detection Research Network Consensus Statement and Standard Operating Procedure Integration Working Group (39). Venous whole blood samples were collected into 10 ml Vacutainer EDTA tubes (BD) via venipuncture after an overnight fast. The tubes were immediately inverted 8–10 times. Blood was processed within 2 h of collection. Samples were centrifuged at 1,300 *g* for 15 min at room temperature. Following centrifugation, the plasma phase was transferred to 1.5 ml DNA LoBind tubes, leaving approximately 1 ml of plasma above the buffy coat, and stored at −80°C.

### Lipoprotein isolation and dialysis

Briefly, VLDLs (density 1.006 g/ml) were removed by spinning plasma at 100,000 *g* for 18 h at 4°C using a fixed-angle rotor (50.2 Ti, Beckman) on an Optima L100 XP ultracentrifuge (Beckman). The VLDL-free plasma was then layered with a 1.063 g/ml KBr solution and centrifuged at 100,000 *g* for 18 h at 4°C. LDLs were isolated and dialyzed first against 0.02 M Trizma, 0.15 M NaCl, 1 mM EDTA, pH 7.5 for 18 h, followed by dialysis against normal saline for 2 h. Finally, isolated LDLs were filter-sterilized using a 0.22 μm Millex-GV filter unit (Millipore). Fresh LDL was used for biochemical and morphometric analyses [dynamic light scattering (DLS) and transmission electron microscopy (TEM)], while lyophilized LDL was used for biophysical studies (FTIR and DSC). Lyophilization was carried out using a vacuum-drying method at room temperature, allowing gradual water evaporation under reduced pressure without freezing. Although this process is slower than conventional freeze-drying, it minimizes potential freezing-induced structural changes in the lipoproteins. After drying, the LDL samples were sent to Toulouse at room temperature for biophysical studies.

### Determination of lipid and apolipoprotein content of LDL

LDL lipid composition, including total and free cholesterol (FC), was determined using enzymatic colorimetric commercial methods adapted for the COBAS 6000/501 autoanalyzer (Roche Diagnostics and Wako Life Sciences). CE content in LDL was calculated as the difference between total and FC. ApoB content in LDL was measured using an immunoturbidimetric assay adapted for the COBAS 6000/501 autoanalyzer (Roche Diagnostics).

The neutral lipid content of CE, TGs, and FC was also determined by TLC following LDL lipid extraction. LDL neutral lipids were extracted using a dichloromethane/methanol [1:2] mixture, and CE, FC, and TG were analyzed on silica G-24 TLC plates, as previously described (40, 41). Standards consisting of a mixture of cholesterol, cholesterol palmitate, and TGs were applied to each plate. Spots corresponding to CE, TG, and FC were identified by densitometry using cholesterol palmitate, TGs, and cholesterol as standards, respectively, in a computing densitometer. Results were expressed as the ratio of CE to FC in each LDL sample.

### Morphometric characterization of LDL particles and SMase-induced LDL aggregates

#### LDL aggregation assay

LDLs isolated from patients with FH and control subjects were concentrated and/or diluted to a final concentration of 0.5 mg/ml in a saline solution. LDLs were incubated with 40 U/L of Bacillus cereus SMase (Sigma-Aldrich, Schnelldorf, Germany) at 37°C for 24 h to induce agLDL. LDLs that were not exposed to SMase for the same duration served as the reference. Lipolysis of LDLs was halted by the addition of EDTA to a final concentration of 10 mM.

#### Dynamic light scattering

Particle size was measured by DLS using the Zetasizer Ultra from Malvern Panalytical. SMase-LDL samples were diluted 1:100, and native LDL samples were diluted 1:20 in PBS. Particle size distribution was determined with the Zetasizer Nano ZS (Malvern Panalytical). Measurements were performed using square polystyrene cuvettes (DTS0012) with a scattering angle fixed at 173° and a temperature of 25°C. Water was used as the dispersant, and each sample was measured in triplicate.

#### TEM of human LDL

TEM was performed using an H-7000 microscope (Hitachi, Japan) with an acceleration voltage of 75 kV. For the analysis, LDL samples were placed on a TEM grid and allowed to settle for 1 min before blotting. The grid was then stained with 2% uranyl acetate for 1 min, after which the excess stain was gently removed using Whatman filter paper. The particle diameters of LDL from the TEM images were quantified using the line tool in ImageJ (v1.54 COQ) to measure length. Subsequently, for each condition (with or without SMase), size distributions were stratified and compared between groups.

### Fourier transform infrared spectroscopy

FTIR/attenuated total reflectance (ATR) spectra of the lyophilized lipoproteins were acquired using a Nicolet 5700 FTIR instrument (Thermo Fisher Scientific, Waltham, MA) equipped with an ATR device with a KBr beam splitter and a MCT/B detector as previously described (42). The ATR accessory used was a Smart Orbit with a type IIA diamond crystal (refractive index 2.4). Freeze-dried samples (1 mg) were directly deposited on the entire active surface of the crystal and gently pressed with a Teflon tip to assure good contact. For each sample, 80 interferograms were recorded in the 4000-450/cm^−1^ region, coadded, and Fourier-transformed to generate an average spectrum of the sample with a nominal resolution between overlapping bands of 2 cm^−1^ using Omnic 8.0 (Thermo Fisher Scientific, Waltham, MA). A single-beam background spectrum was collected from the clean diamond crystal before each experiment, and this background was subtracted from the spectra. Spectra were then subjected to ATR and rubber-line baseline corrections and normalized using the maximum of the amide II peak. Second derivatives were used to enhance the chemical information present in overlapping infrared absorption bands of spectra. Mean spectra from each category of human LDLs were generated to obtain a clearer representation, and main bands were identified according to literature data (Table 2). For quantitative analysis, the area of bands of interest were determined from each individual spectrum, and the appropriate area ratios proportional to the amount of the different components were generated. The decomposition of the amide I was performed to quantify the relative proportion of protein secondary structures (43, 44, 45, 46) using the Peak Resolve function of the Omnic 8.0 Software. Based on the characteristic minima of the second derivative curves, the spectral range of 1600–1700 cm^−1^ was decomposed in 8 bands whose positions are allowed to range between fixed limits (Table 3). In the curve-fitting procedure, the combination of Gaussian–Lorentzian peak shape was used for all the peaks, with a full-width-half-height ranging between 5 and 30 cm^−1^. A proportion of each component in the amide I band was computed as a fractional area of the corresponding peak divided by the sum of the areas of the peaks belonging to the amide I band.

**Table 2:** Main FTIR absorption bands in human LDL

Position (cm^−1^)	Assignment
3394, 3276	Amide A, mainly the ν(N-H) mode of proteins with the contribution of the ν(O-H) stretching mode of H_2_O
3076	Amide B first overstone of amide II
3009	ν(C=H) of unsaturated lipids
2952, 2922, 2866, 2851	ν_as_(CH_3_) of proteins and lipids, ν_as_(CH_2_) of lipids ν_s_(CH_3_) of proteins and lipids,ν_s_(CH_2_) of lipids
1736	ν(C=O) of esterified lipids (mainly phospholipids, CE, and TG)
1718–1714 (shoulder)	ν(C=O) of free fatty acids
1640–1623	Amide I: mainly ν(C=O) of proteins
1545	Amide II: ν(C-N), δ (N-H) of proteins
1464, 1456	δ(CH_2_) scissoring, δ(CH_3_) bending of lipids and proteins
1365	CH_2_ wagging, overlapping with protein band B
1280	Amide III of proteins
1253	Amide III of proteins and ν_as_(PO_2_^-^) of phospholipids
1171	ν_as_(CO-O-C) of esters
1085	ν_s_(PO_2_^-^ symmetric stretching ν_s_(C-O-C) of esters
1057	
973	ν_as_(N+(CH_3_)_3_) of choline groups in phospholipids
925	skeletal ν(C-C) or ν(C-C) from phospholipids’ head
800	δ(C-H) bending of CE
720, 700	Rocking band of methylene groups in lipids

CE, cholesteryl ester; FTIR, Fourier transform infrared spectroscopy; TG, triglyceride.

**Table 3:** Curve-fitting analysis of amide I band from FTIR spectra, mainly corresponding to C=O stretching vibrations in ApoB 100 and highly sensitive to secondary structures

Peak Number	Assignment	FWHH (cm^−1^)	Accepted Limits (cm^−1^)	Shape
1	Side chain	5–30	1605–1617	Gaussien/Lorentzien
2	Intermolecular β sheets, β strands	5–30	1618–1625	Gaussien/Lorentzien
3	Intramolecular β sheets	5–30	1630–1636	Gaussien/Lorentzien
4	Random coil/water	5–30	1640–1646	Gaussien/Lorentzien
5	Random coil/α helices	5–30	1648–1652	Gaussien/Lorentzien
6	α helices	5–30	1657–1662	Gaussien/Lorentzien
7	Turns, loops	5–30	1665–1672	Gaussien/Lorentzien
8	Turns, β sheets	5–30	1678–1690	Gaussien/Lorentzien

FTIR, Fourier transform infrared spectroscopy; FWHH, full-width-half-height.

### Thermal analysis by DSC

We used lyophilized LDL to ensure that the DSC measurements accurately reflected the intrinsic thermal properties of the LDL components, avoiding interference from extraneous factors such as water or degradation. Calorimetric analyses of lyophilized LDL samples were performed using a DSC Q2000 calorimeter (TA Instruments, Waters, New Castle, DE). The calorimeter was calibrated with cyclohexane and indium as standards, ensuring a temperature accuracy of ±0.1°C and an enthalpy accuracy of ±0.2 J/g. LDL samples weighing 5–10 mg were placed in nonhermetic aluminum pans and equilibrated at the initial temperature for 5 min before being cooled to −120°C at a rate of 10°C/min. Thermograms were then recorded during heating at a rate of 10°C/min until the temperature reached 120°C. Thermal transitions were analyzed using TA Instruments Universal Analysis software (version 4.5). First-order transitions, identified as peaks in the DSC thermograms, were characterized by the temperature at the peak maximum, the peak area, and the width at half-height of the peak.

### Statistical analysis

All results are presented as scatter plots with means ± SD. Statistical analysis was performed using Prism software (version 9.0, GraphPad). Normal distribution of all variables was assessed using the Shapiro–Wilk normality test. The Kruskal–Wallis test, followed by Dunn’s posthoc test, was employed when one or more groups did not exhibit Gaussian distribution. For comparisons between two groups, the unpaired two-tailed Student’s *t* test (for unequal variances) or two-way ANOVA followed by Tukey’s posthoc test (for normality and variance homogeneity) was used. Differences were considered statistically significant at *P* <0.05.

To analyze potential relationships between quantitative variables, the Spearman’s correlation coefficient (r) matrix was computed using OriginPro 2023 (version 10.0.0154, OriginLab Corporation, Northampton, MA). This coefficient matrix was visualized as a correlogram, where the color represents the value of the Spearman’s coefficient and the shape corresponds to the confidence ellipse of the scatter plot between variables. Significant correlations (*P* values <0.05, 0.01, 0.001) were indicated with asterisks.

To assess the feasibility of DLS for evaluating the size of single LDL particles and SMase-induced agLDL, as well as the fold change induced by SMase, we measured the average coefficient of variation (CV) from the means of pooled LDL in each of the three measurement rounds.

## Results

### Biochemical differences in LDL from patients with FH and control subjects

**Fig. 1:**
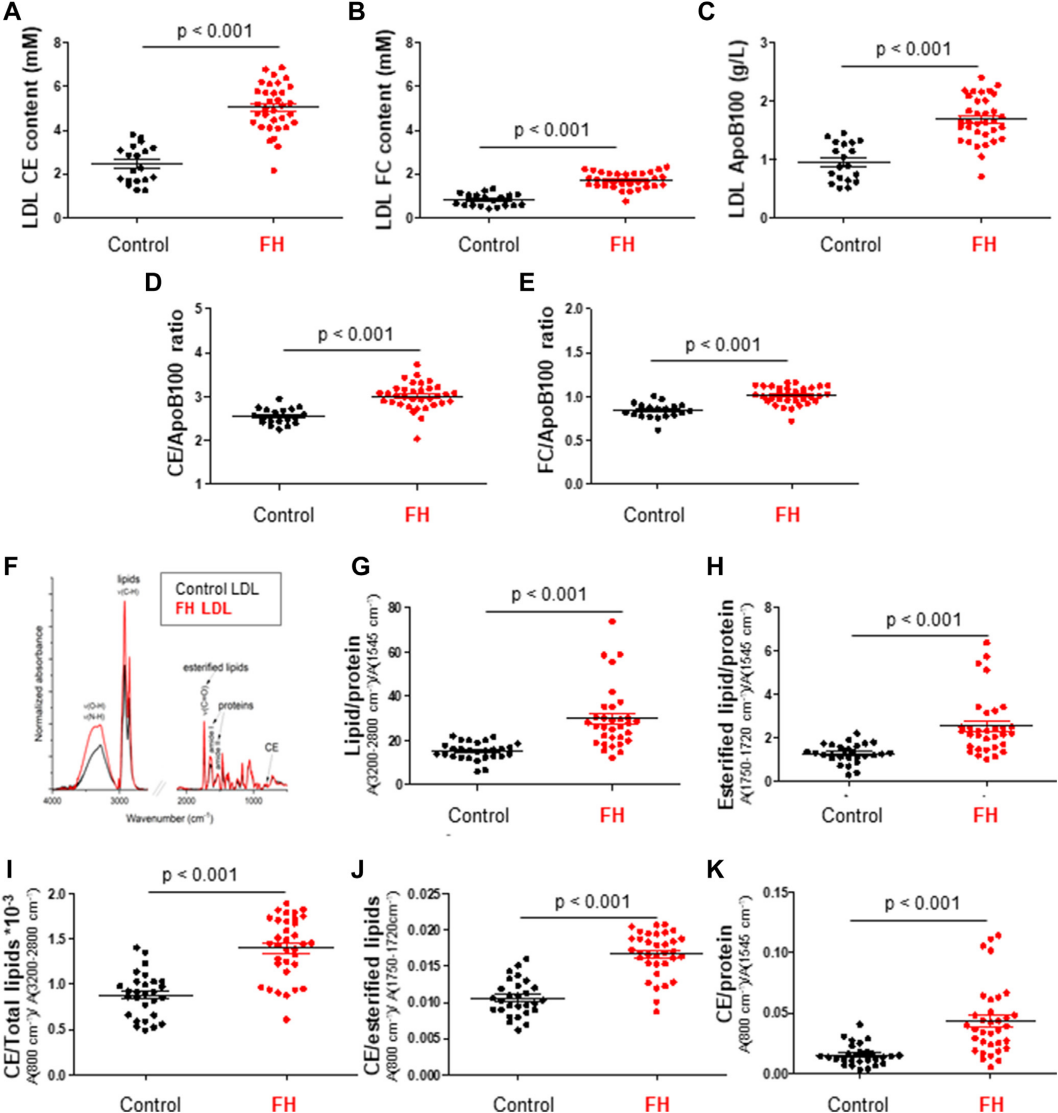
Lipids in LDL from patients with FH compared to LDL from control subjects. Biochemical determinations in LDL including cholesteryl ester (CE) content (A), free cholesterol content (B), ApoB100 (C), CE/ApoB100 ratio (D) and FC/ApoB100 ratio (E). FTIR spectra showing the normalized absorbance peaks and their molecular assignments in the 4000-500 cm⁻^1^ wavelength range in lyophilized human LDL (F). Graphs displaying differences in the indicators of total lipids/proteins (band area (3200–2800 cm⁻^1^) normalized by band area at 1545 cm⁻^1^) (G), esterified lipids/proteins (band area (1750–1720 cm⁻^1^) normalized by band area at 1545 cm⁻^1^) (H), cholesteryl esters/proteins (band area (804–796 cm⁻^1^) normalized by band area at 1545 cm⁻^1^) (I), cholesteryl esters/total lipids (band area (804–796 cm⁻^1^) normalized by band area (3200-2800 cm⁻^1^) (J), and cholesteryl esters/esterified lipids (band area (804–796 cm⁻^1^) normalized by band area (1750–1720 cm⁻^1^) (K). Data are shown as mean ± SD. For comparison of two groups, the unpaired two-tailed Student’s *t* test (for unequal variances) or two-way ANOVA followed by Tukey’s posthoc test (for normality and variance homogeneity) was used. Differences were considered statistically significant when *P* < 0.05. FH, familial hypercholesterolemia; FTIR, Fourier transform infrared spectroscopy; Stds, standards.

In this study, we initially conducted biochemical analyses on serum samples from 35 patients with FH (FH) and 29 healthy control subjects. Table 1 provides a summary of the general lipid characteristics for both groups. Notably, biochemical results revealed significantly elevated levels of total serum cholesterol, LDL-C, and apolipoprotein B (ApoB), in FH patients compared to control subjects (*P* < 0.001 for all comparisons). Regarding LDLc, the content of CE (CE) (Fig. 1A), FC (FC) (Fig. 1B), and TGs (online supplemental Fig. S1A), as well as ApoB100 levels (Fig. 1C), were significantly increased in LDL from FH patients compared to control subjects (*P* < 0.001 for all comparisons). Additionally, both the CE/ApoB100 (Fig. 1D) and FC/ApoB100 (Fig. 1E) ratios were markedly elevated in FH patients compared to controls. In contrast, the TG/CE ratio (online supplemental Fig. S1B) showed no significant difference between FH and controls (*P* = 0.528). These findings clearly indicate a higher LDL particle number and an increased cholesterol content per LDL particle in FH patients.

### Differences in FTIR signature of lipids in LDL from patients with FH and control subjects

We performed a biophysical characterization of LDL using FTIR spectroscopy. FTIR spectroscopy measures the interaction between infrared radiation and the covalent bonds of molecules, which vibrate at specific frequencies corresponding to distinct energy levels (vibrational modes). In the averaged FTIR spectra of freeze-dried LDL (Fig. 1A), prominent absorption bands were detected in the [3050–2800 cm⁻^1^] range, primarily originating from lipids. In this infrared region, lipoproteins exhibit specific absorption bands for asymmetric and symmetric (CH₃) and (CH₂) stretching, which are primarily associated with the vibrational modes of various lipid classes. Additionally, the (C=H) stretching at 3010 cm⁻^1^ corresponds to unsaturated lipids. A notable and intense absorption is found in the [1745–1735 cm⁻^1^] range, corresponding to (C=O) stretching from esterified lipids (47). A weaker band for CE absorption is detected at 800 cm⁻^1^ (42). As reported previously (45, 48, 49), the two broad bands in the [1700–1500 cm⁻^1^] range, known as amide I and amide II, mainly contain C=O stretching and NH bending/CN stretching of protein peptide bonds, respectively, serving as the spectral signature of ApoB100 protein in LDL (45). The main band assignments are summarized in Table 2. The ratios of band areas associated with lipid indicators were calculated from each individual spectrum and were significantly higher in LDL from FH patients compared to controls. These lipid indicators include total lipids/proteins (CHx/amide II, *P* < 0.001) (Fig. 1F, G), esterified lipids/proteins (CO ester/amide II, *P* < 0.001) (Fig. 1F, H), CE/total lipids (CE/CHx, *P* < 0.001) (Fig. 1I), CE/esterified lipids (CE/CO ester, *P* < 0.001) (Fig. 1J), and CEs (CE)/proteins (CE/amide II, *P* < 0.001) (Fig. 1K). Additionally, TLC of LDL lipid extracts demonstrated a significant increase in the CE/FC ratio in LDL from FH patients compared to controls (*P* < 0.001) (online supplemental Fig. S2). Statistical analysis demonstrated a strong correlation between biochemical CE/ApoB100 and vibrational CE/amide II variables in LDL (r = 0.479, *P* < 0.001).

### Differences in the thermal profile of LDL from patients with FH and control subjects

In the literature, DSC has been used to study thermal characteristics of lipoproteins in various states, including freeze-dried, precipitated, highly concentrated, or diluted (ranging from 2 g/ml to 1 mg/ml) (50, 51, 52, 53, 54, 55). Here, lipoprotein thermal transitions were analyzed in lyophilized LDL for three main reasons: 1) to avoid variations in hydration in the highly concentrated state, which can affect the parameters of thermal transitions; 2) to extend the temperature exploration range below 10°C, a zone where certain lipids undergo thermal transitions that cannot be observed in the diluted state; and 3) to compare the thermal transitions of LDL with those of anhydrous cholesterol esters and other lipids referenced in the literature.

**Fig. 2:**
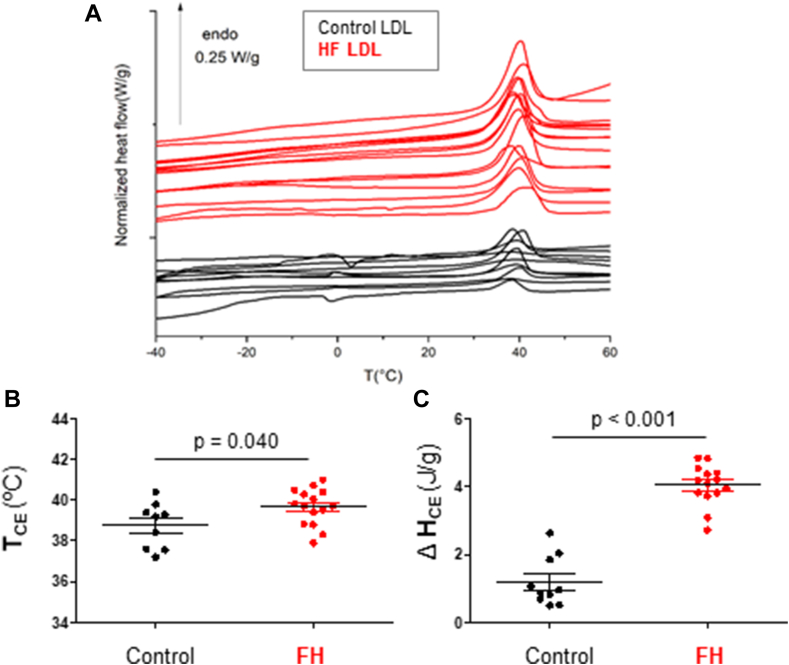
Thermal profile of LDL from patients with FH compared to LDL from control subjects. A: Differential scanning calorimetry (DSC) thermograms of human LDL showing the main endothermic peak in the 30–50°C range, which reflects the smectic-to-disorder phase transition of the lipid CE core. Grouped scatter plots comparing the transition temperature of the CE core (T_CE_) (B) and the normalized enthalpy of the transition associated with the area of the peak (ΔH_CE_) (C) in LDL from control subjects and patients with FH. Data are shown as mean ± SD. For comparison of two groups, the unpaired two-tailed Student’s *t* test (for unequal variances) or two-way ANOVA followed by Tukey’s posthoc test (for normality and variance homogeneity) was used. Differences were considered statistically significant when *P* < 0.05. CE, cholesteryl ester; FH, familial hypercholesterolemia.

DSC thermograms of human LDL are characterized by a main endothermic peak in the 30–50°C range, reflecting the smectic-to-disorder phase transition in the CE core (Fig. 2A), consistent with previous studies on human plasma LDL (51, 52, 53, 54, 55). The transition temperature of the CE core (T_CE_) in LDL was shifted to higher values for FH patients than controls (*P* = 0.040) (Fig. 2B). Additionally, the normalized enthalpy of the transition, associated with the area of the peak (ΔH_CE_), was significantly increased in LDL from FH patients compared to controls (*P* < 0.001) (Fig. 2C). The width at half-height of this peak, an indicator of the cooperativity of the CE transition, showed no significant difference (*P* = 0.536) between LDL from FH patients (6.0 ± 0.7°C) and controls (6.4 ± 1.62°C).

Statistical analysis revealed strong correlations between biochemical, vibrational, and thermal data of LDL. Specifically, the cholesterol/ApoB protein ratio significantly correlated with CE/amide II (r = 0.692, *P* < 0.001) (online supplemental Fig. S3A), with ΔHCE (r = 0.736, *P* < 0.001) (online supplemental Fig. S3B), and with T_CE_ (r = 0.557, *P* < 0.005) (online supplemental Fig. S3D). Additionally, ΔHCE strongly correlated with the cholesterol/protein ratio (r = 0.804, *P* < 0.001) (online supplemental Fig. S3C) and with T_CE_ (r = 0.556, *P* < 0.005) (online supplemental Fig. S3E).

### Differences in the relative number of ApoB100 secondary structures in LDL from patients with FH and control subjects

**Fig. 3:**
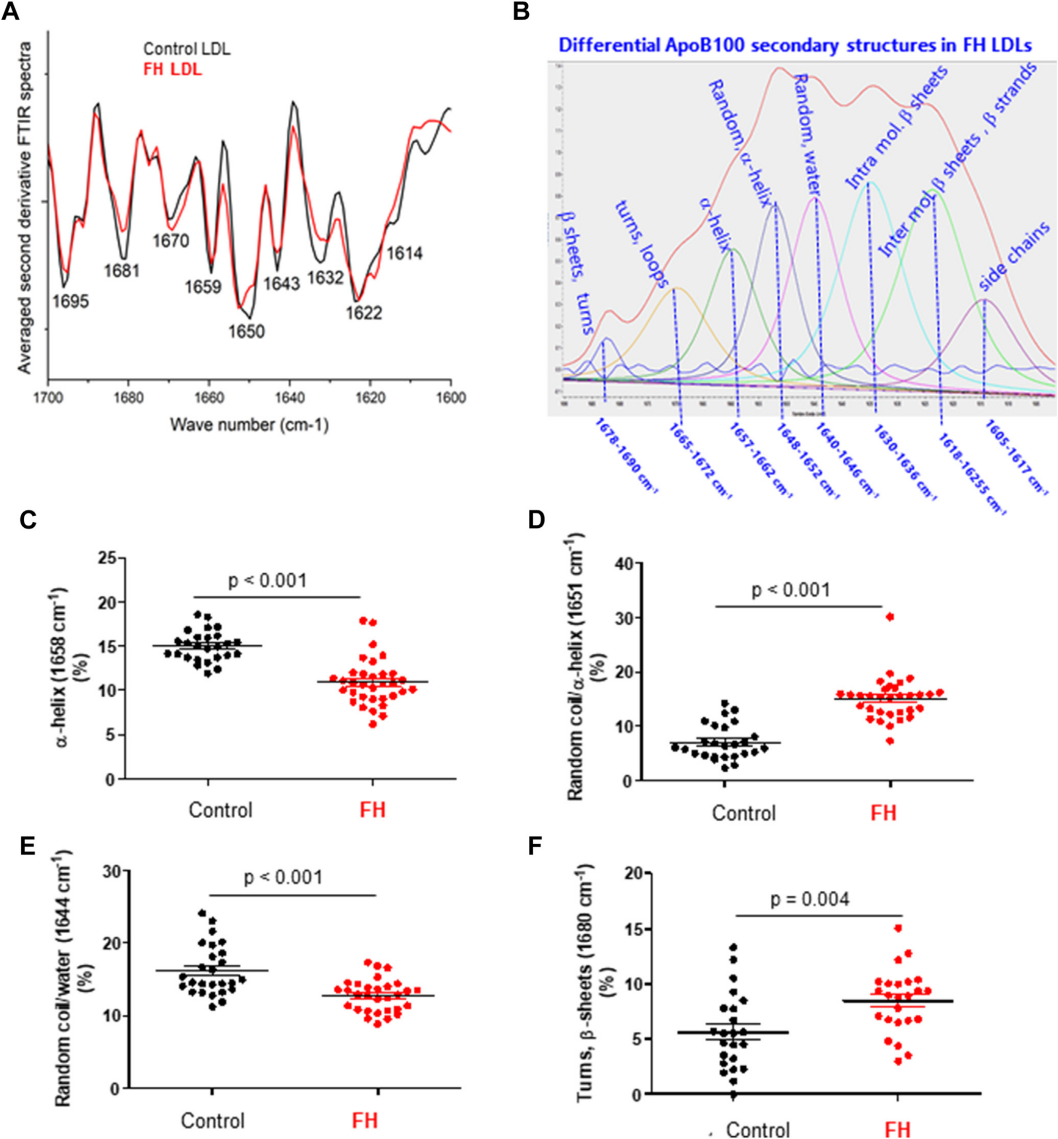
FTIR signature of secondary structures in ApoB100 (LDL) from patients with FH compared to LDL from control subjects. A: comparison of representative second derivative FTIR spectra in LDL from patients with FH and control subjects in the 1700–1600 cm⁻^1^ range corresponding to the amide I band. Marked minima indicate contributions from secondary structures of ApoB100 in human LDL. B: Representative decomposition of the amide I band according to the curve-fitting procedure detailed in Table 3, with the assignment of secondary structures. Grouped scatter plots showing the percentage of the α-helices (1658 cm⁻^1^) component (C), the α-helices and random coil (1651 cm⁻^1^) component (D), the random coil/water (1644 cm⁻^1^) component (E), and the turns/β-sheets (1680 cm⁻^1^) component (F) in each tested group. Data are shown as mean ± SD. For comparison of two groups, the unpaired two-tailed Student’s *t* test (for unequal variances) or two-way ANOVA followed by Tukey’s posthoc test (for normality and variance homogeneity) was used. Differences were considered statistically significant when *P* < 0.05. FH, familial hypercholesterolemia; FTIR, Fourier transform infrared spectroscopy.

FTIR allows the identification of different protein secondary structures, particularly using the amide I region (43, 44, 46, 56). As noted in these studies, the amide I vibration, absorbing near 1650 cm⁻^1^, primarily arises from the C=O stretching vibration of the protein peptide bond, with minor contributions from the out-of-phase CN stretching vibration, CCN deformation, and NH in-plane bending (43, 44, 46, 56). The differences in the frequencies of α-helices, β-sheets, β-turns, and unordered structures in the amide I band of FTIR spectra are attributed to variations in hydrogen bonding, dipole interactions, and the structural arrangement among these distinct secondary structures. The second derivative of the FTIR spectra, which enhances resolution, was performed in the 1700–1600 cm⁻^1^ spectral zone, assigned to the C=O stretching vibration of ApoB 100 peptide bond amide I mode. The averaged second derivative FTIR spectra of LDL from controls and patients with FH revealed distinct minima in this region (Fig. 3A), suggesting contributions from various types of secondary structures in the ApoB100 protein for both patient and control LDL. The main secondary structures present in the ApoB100 protein from human LDL are β-structures, including β-strands (1622, 1694 cm⁻^1^) embedded in the LDL monolayer, α-helices (1658, 1651 cm⁻^1^), random coils (1643 cm⁻^1^, 1651, 1643 cm⁻^1^), β-sheets (1632 cm⁻^1^), and β-turns (1681, 1670 cm⁻^1^), as previously reported in the literature (45, 57, 58). The relative proportion of protein secondary structures can be evaluated from the decomposition of the amide I band performed for each individual spectrum. This quantification of secondary structures revealed specific differences in ApoB100 secondary structures of LDL between FH patients and control subjects. Notably, the two components at 1658 and 1651 cm⁻^1^, which correspond to α-helical structures, were inversely altered in FH. Both peaks (1651 cm⁻^1^ and 1658 cm⁻^1^) can be attributed to carbonyl groups, but the specific context influences their exact wavenumber. These peaks are indicative of the molecular environment surrounding the carbonyl group, providing insights into the structural characteristics of ApoB100. While the component at 1658 cm⁻^1^ is unambiguously attributed to α-helices, both α-helices and unordered structures can contribute to the 1651 cm⁻^1^ component in nondeuterated solutions or freeze-dried proteins (35, 43, 44). In FH patients compared to controls, the percentage of the 1658 cm⁻^1^ component decreased (*P* < 0.001) (Fig. 3A, B and C), whereas the percentage of the 1651 cm⁻^1^ component increased (*P* < 0.001) (Fig. 3A, B and C). The presence of both 1658 cm⁻^1^ and 1651 cm⁻^1^ components in the absorption zone of C=O stretching vibration of α helical protein structures has been observed in other types of proteins (35, 46). Since factors such as hydrogen bonding, increased helix length, and helix packing can lower the absorption frequency of the C=O stretching vibration (44), the observed splitting into two components has been attributed to dynamic and flexible helices at the higher frequency, and more stable helices at the lower frequency. We relied on these attributions to explain the coexistence of two components in the 1650–1660 cm^−1^ zone and the possible modification of the alpha-helical properties of ApoB100 in LDL from HF patients compared to control.

There were also changes in the amounts of other secondary structures in human ApoB100 in FH patients compared to controls. Specifically, the random coil/water component at 1644 cm⁻^1^ was reduced in LDL from FH patients versus controls (*P* < 0.001) (Fig. 3A, B, and E). Conversely, the turn and β-sheet component at 1680 cm⁻^1^ was increased in LDL from FH patients (*P* = 0.004) (Fig. 3A, B, and F). The percentages of other components, such as β-strands embedded in the LDL monolayer (1622 cm⁻^1^) (*P* = 0.115) (online supplemental Fig. S4A), intramolecular β-sheets (1633 cm⁻^1^) (*P* = 0.802) (online supplemental Fig. S4B), and turns/loops (1668 cm⁻^1^) (*P* = 0.058) (online supplemental Fig. S4C), did not show significant differences between the groups.

**Fig. 4:**
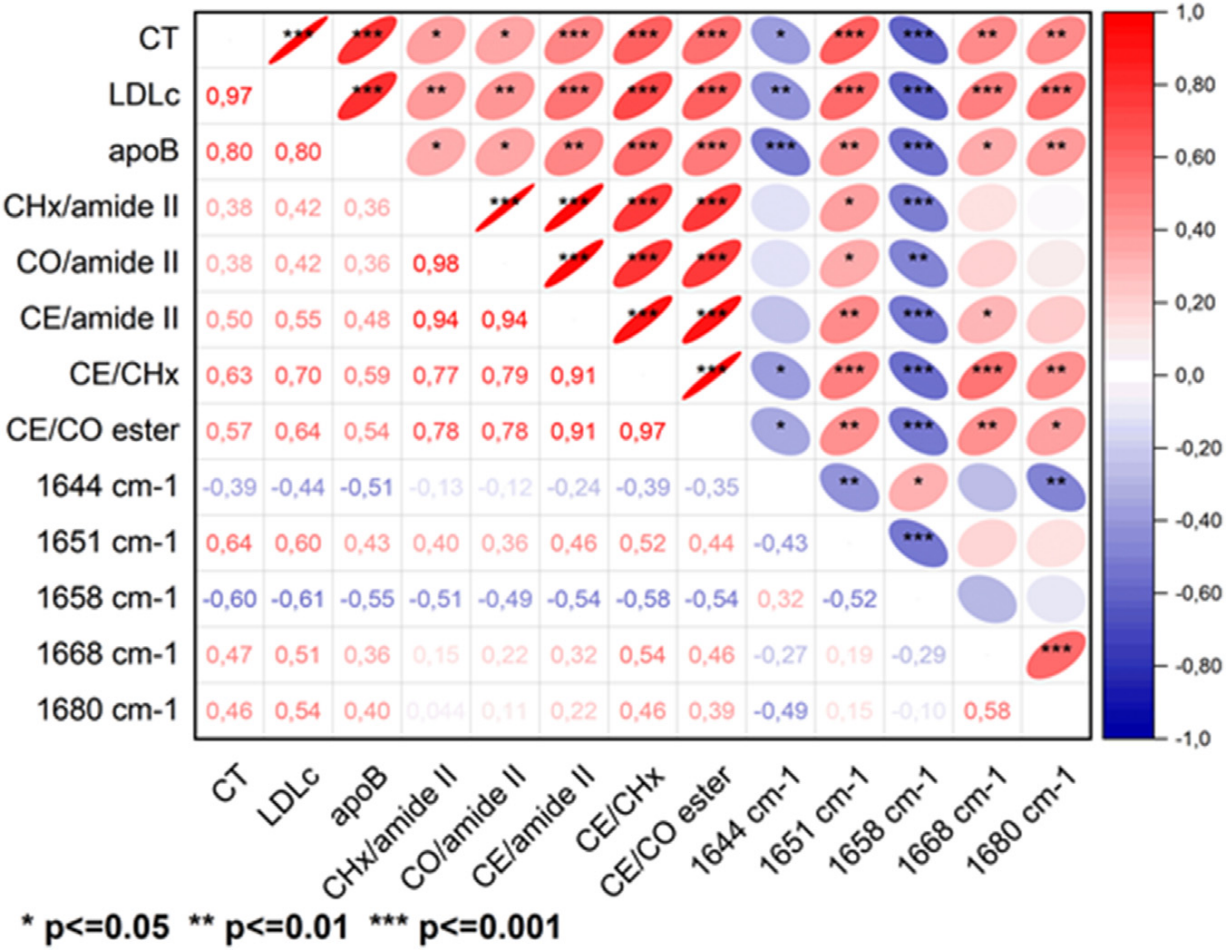
Correlogram showing the significant correlations between LDL-CE composition, and the percentage of secondary structures in ApoB100. Positive correlations are shown in red and negative correlations in blue, with the intensity of the color representing the Spearman’s correlation coefficient. The shape of each cell corresponds to the confidence ellipse of the scatter plot between variables. Significant levels are indicated with asterisks. CE, cholesteryl ester.

The correlogram (Fig. 4) revealed that biochemical and biophysical lipid variables related to LDL cholesterol, particularly LDL CEs (CE/amide II, CE/CHx, CE/CO ester), directly correlated with the 1651 cm⁻^1^ component (stable α-helix/random coil) (*P* < 0.01) and inversely with the 1658 cm⁻^1^ component (flexible α-helix) (*P* < 0.001). Additionally, CE/CHx and CE/CO ester directly correlated with the percentage of the 1680 cm⁻^1^ component (β-sheet) (*P* < 0.01 and *P* < 0.05, respectively).

### Differences in LDL susceptibility to aggregation in patients with FH compared to control subjects

According to results from the human LDL pool used as an internal control across the three sets of patient measurements, DLS is a reliable and reproducible technique for measuring changes in LDL size and distribution. The inter-assay CV for unmodified and SMase-induced agLDL was 6% and 7%, respectively. Additionally, the CV for the fold change in Z-average LDL particle size induced by SMase was 2% (online supplemental Fig. S5), validating the fold change as a suitable indicator of LDL susceptibility to aggregation.

**Fig. 5:**
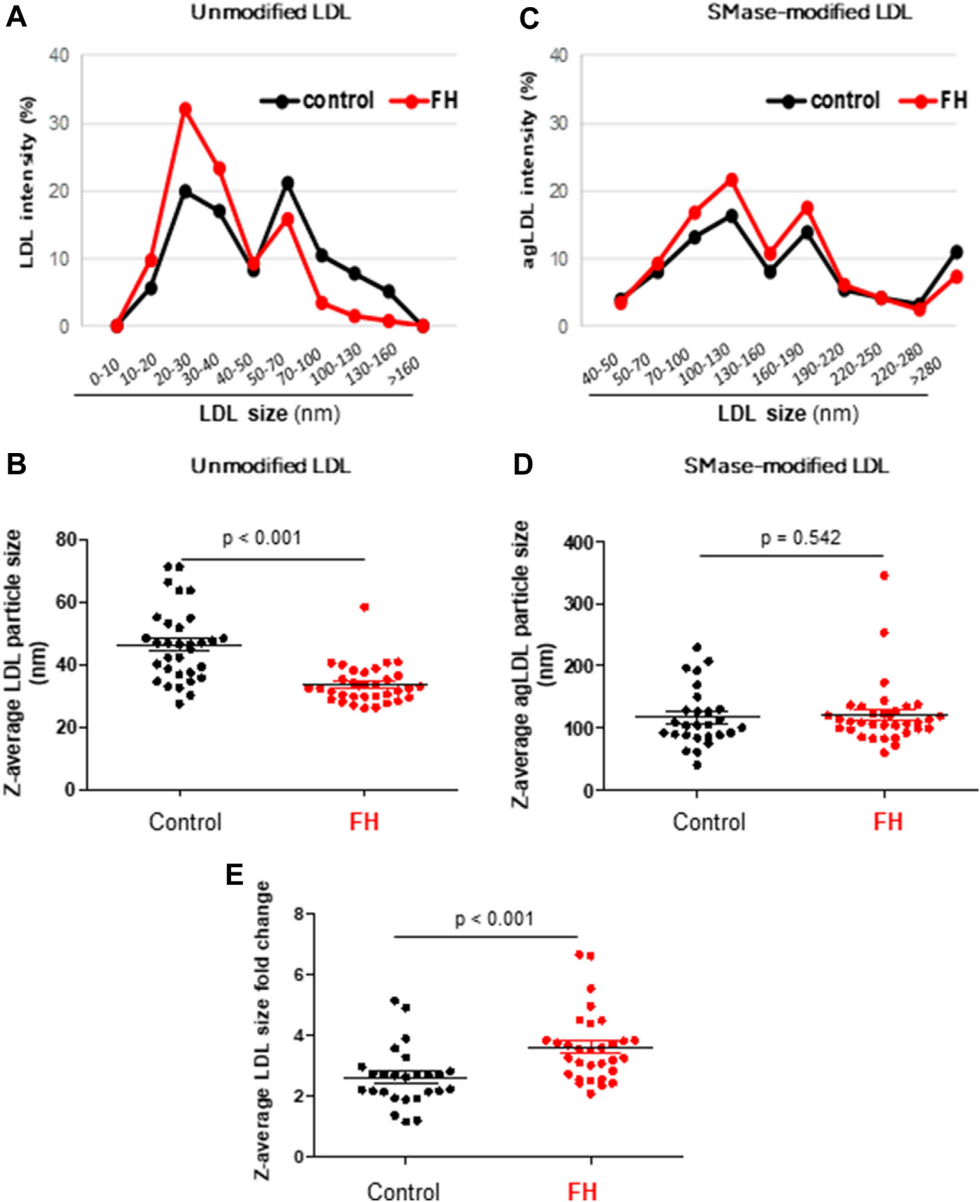
Differences in the particle size distribution and the Z-average LDL particle size measured by DLS in unmodified LDL, SMase-modified LDL, and fold change between patients with FH and control subjects. LDL particle size distribution in LDL from patients with FH and control subjects was analyzed by dynamic light scattering (DLS) measurements in unmodified LDL and SMase-induced aggregated LDL. Graphs illustrate the distribution and Z-average LDL size of unmodified (A, B) and of SMase-induced LDL aggregated LDL (C, D) particle size. The fold change in the Z-average size between aggregated and unmodified LDL in each patient was used to define individual susceptibility of LDL to aggregation (C). Z-average and fold change data are shown as the mean ± SD of LDL particles isolated from control subjects (n = 29) and patients with FH (n = 35). For comparison of two groups, the unpaired two-tailed Student’s *t* test (for unequal variances) or two-way ANOVA followed by Tukey’s posthoc test (for normality and variance homogeneity) was used. Differences were considered statistically significant when *P* < 0.05. FH, familial hypercholesterolemia; SMase, sphingomyelinase.

**Fig. 6:**
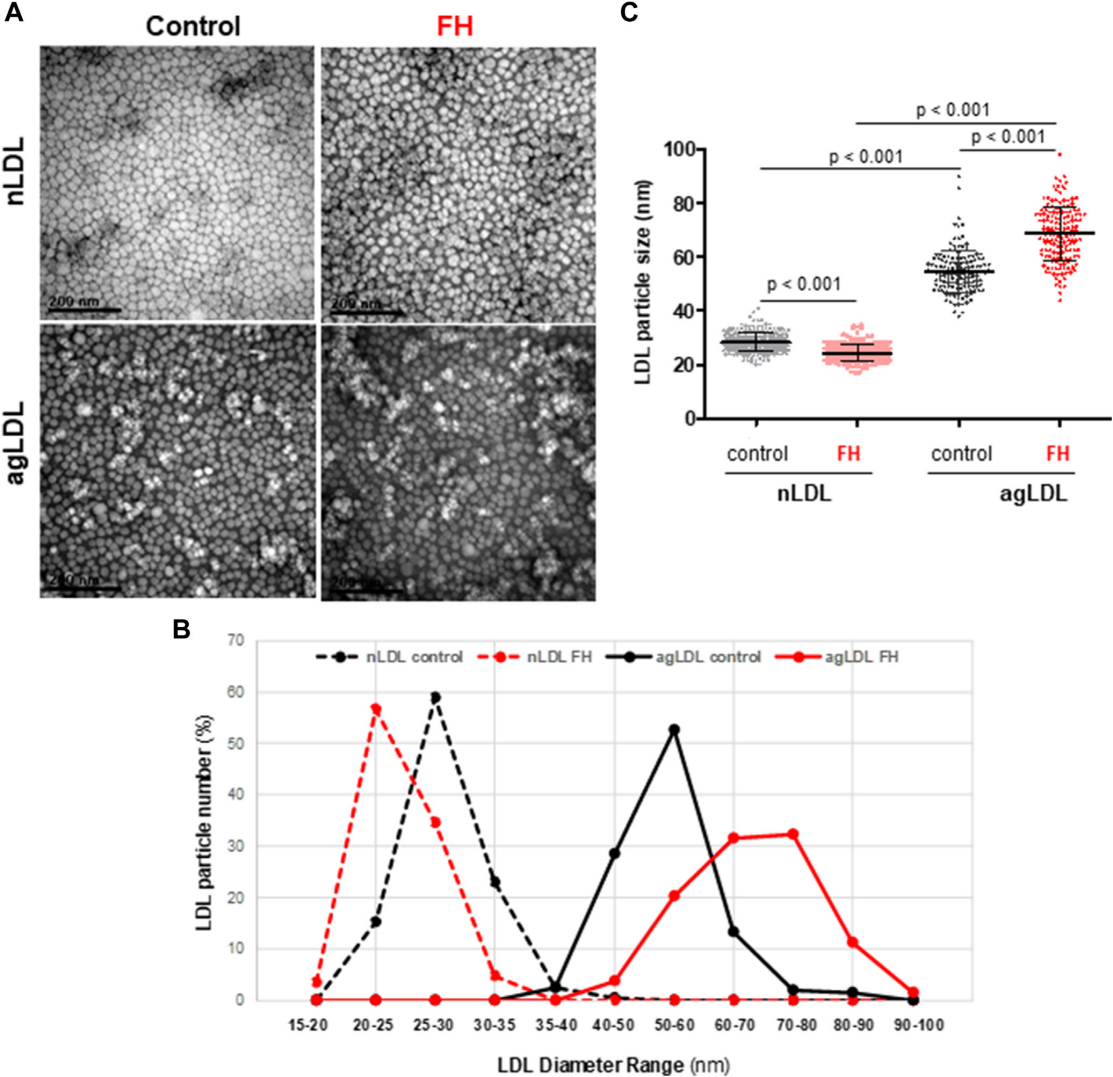
LDL particle diameter and LDL size distribution in unmodified LDL and SMase-induced LDL aggregates in patients with FH and control subjects. Representative TEM images (A) and line graphs (B) illustrate the morphology and size distribution of unmodified LDL (nLDL) and aggregated LDL (agLDL) particles. The size distribution of LDL particles is presented as the percentage of particles within each diameter range, relative to the total number of particles in each TEM section (20–28 particles per section). C: graphs showing the comparison of unmodified and aggregated LDL (agLDL) particle size. Data are shown as mean ± SD. For comparison of two groups, the unpaired two-tailed Student’s *t*-test (for unequal variances) or two-way ANOVA followed by Tukey’s posthoc test (for normality and variance homogeneity) was used. Differences were considered statistically significant when *P* < 0.05. FH, familial hypercholesterolemia; TEM, transmission electron microscopy.

In reference to unmodified LDL, DLS analysis revealed a bimodal distribution of unmodified LDL particle intensity, suggesting the presence of at least two distinct populations of LDL particles in both FH and control subjects (Fig. 5A). The percentage of particles in the 10–50 nm range was significantly higher in FH subjects than in controls (74.54 ± 13.76% versus 47.20 ± 16.16%, *P* < 0.001), whereas the percentage of particles in the 51–160 nm range was lower in FH subjects compared to controls (21.56 ± 11.41% versus 74.54 ± 13.76%, *P* < 0.001). Similarly, the Z-average particle size of LDL in FH patients was significantly smaller than in control subjects (*P* < 0.001) (Fig. 5B). Consistent with these findings, particle size distribution analysis using TEM showed that 56.73 ± 9.34% of particles in FH patients fell within the 20–25 nm range, while in controls, 56.84 ± 5.46% of particles were in the 25–30 nm range (Fig. 6A, B). Additionally, TEM indicated that the mean LDL particle diameter in FH patients was 24.36 ± 3.05 nm, compared to 28.31 ± 3.38 nm in controls (*P* < 0.001) (Fig. 6A, C).

In reference to SMase-modified LDL, DLS analysis also revealed a bimodal distribution of LDL particles exposed to SMase (agLDL) in both FH and control LDL samples (Fig. 5C). The percentage of agLDL particles within the 30–160 nm size range was significantly higher in FH subjects than in controls (61.88 ± 17.85% *versus* 49.64 ± 24.90%, *P* = 0.029), while no significant differences were observed in the percentage of particles larger than 160 nm between the groups (*P* = 0.968). The Z-average particle size of LDL exposed to SMase (agLDL) did not differ significantly between FH patients and controls (*P* = 0.542) (Fig. 5D). However, the fold change in Z-average particle size between agLDL and unmodified LDL was significantly greater in FH patients than in controls (*P* < 0.001) (Fig. 5E).

TEM analysis also revealed differences in the size distribution of agLDL particles between patients and controls (Fig. 6A, B). AgLDL from control subjects showed a sharp peak, with most particles falling within the 50–60 nm diameter range, while LDL from FH patients displayed a broader, flatter peak, with the majority of particles distributed within the 60–80 nm range (Fig. 6C). Consistent with this, TEM indicated an agLDL particle diameter of 68.67 ± 10.19 nm in FH patients, compared to 54.58 ± 8.00 nm in controls (*P* < 0.001) (Fig. 6A, C).

**Fig. 7:**
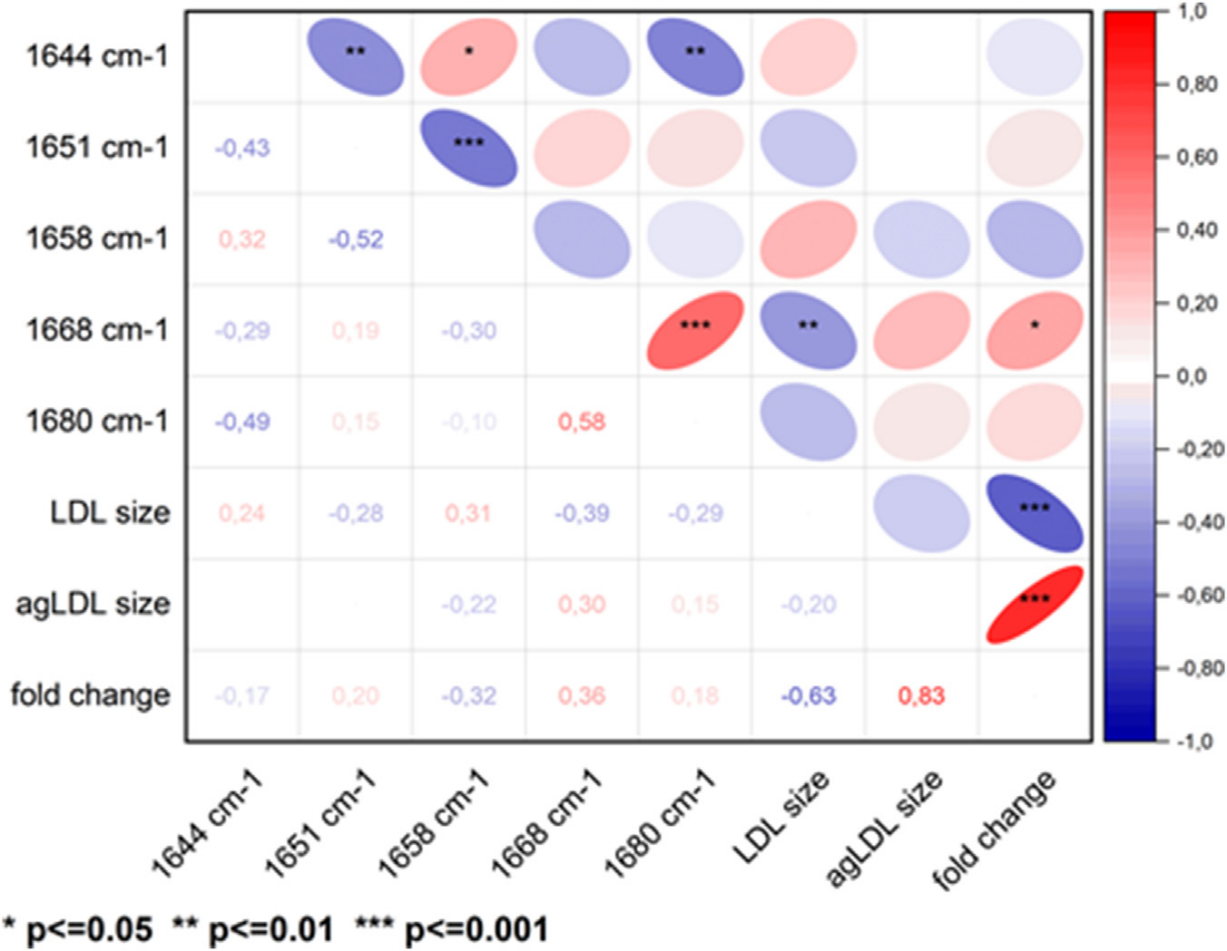
Correlogram showing the significant correlations between the percentage of secondary structures in ApoB100, and susceptibility of LDL to aggregation. Positive correlations are shown in red and negative correlations in blue, with the intensity of the color representing the Spearman’s correlation coefficient. The shape of each cell corresponds to the confidence ellipse of the scatter plot between variables. Significant levels are indicated with asterisks.

The correlogram (Fig. 7) based on DLS measurements illustrates the correlations between LDL morphometry and various biochemical/biophysical variables. It shows that CE/CHx and CE/CO ester were inversely correlated with LDL size (*P* = 0.029 and *P* = 0.020, respectively), but positively correlated with agLDL size (*P* = 0.004 and *P* = 0.002, respectively) and the fold change in LDL size (*P* = 0.010 and *P* = 0.007, respectively). Additionally, the percentage of flexible α-helix (1658 cm⁻^1^) was inversely correlated with the fold change in LDL size (*P* = 0.028)

## Discussion

This study reveals several key findings regarding LDL from patients with FH. First, we observed that increased CE content in LDL from FH patients is associated with a reduction in LDL size. Second, this increase in CE content correlates with heightened thermal stability of the ordered CE-core. Third, we identified differences in the pattern of ApoB100 secondary structures in LDL from FH patients compared to controls.

Notably, our findings demonstrate for the first time that ApoB100 in FH patients exhibits a decreased proportion of flexible α-helix structures. This reduction in flexible α-helices is likely a contributing factor to the increased susceptibility of LDL to aggregation observed in FH patients.

The reduction in LDL size observed in patients with FH using DLS and TEM techniques is likely attributable to the compacting effect of CEs on the spherical structure of LDL particles. From a biophysical perspective, the chemical and biophysical properties of CE play a critical role in lipid packaging and lipoprotein nucleation (59). This compacting effect of CE has been previously documented in lipid droplets (LDs) of cardiomyocytes from rabbits fed a high-fat diet (42). Unlike the results obtained in this study, previous studies have reported either larger, more buoyant LDL particles (60, 61, 62) or similar-sized particles (63) in FH patients compared to controls. The differences in LDL particle size observed in our study, compared to previous reports, could be attributed, at least in part, to the distinct methodologies used for particle size determination. Our study employed DLS and TEM, whereas prior studies utilized techniques such as nondenaturing polyacrylamide gradient gels (60) or NMR spectroscopy (63). TEM is widely regarded as one of the most precise methods for assessing particle size and morphology (64), while DLS has been recognized as a highly effective technique for measuring particle size distribution in colloidal systems, particularly for particles ranging from 1 nm to 1 μm (65). An additional factor contributing to the observed differences in LDL particle size between our study and previous studies, such as that by Paiker *et al.*, may stem from variations in the control and FH subject populations. The participants in the Paiker *et al.* study were of African descent, whereas those in our study are of European descent. Ethnic differences in LDL particle size have been suggested to contribute to the disparities in CVD prevalence across different populations (66).

Here, DSC results indicated that the endothermic transition in human LDL, corresponding to the order-disorder transition in the CE core, occurs within the 37–41°C range. This transition has been reported to vary between 26 and 40°C depending on diet, with a shift toward higher temperatures associated with high-fat diets (54) or a decrease in TGs (51, 67). Notably, our results showed that the smectic-to-disorder phase transition in the CE core of LDL was shifted toward higher temperatures in FH patients than in controls, indicating that the ordered phase is more persistent at physiological temperatures in these patients. Increased values for this phase transition have also been observed in CE-enriched LDs and have been directly attributed to elevated CE levels (68). The authors propose that CEs induce a liquid-crystalline phase in LDs, which in turn affects the distribution of proteins associated with the LD surface.

Results from the second derivative of the FTIR spectra in the 1700–1600 cm⁻^1^ spectral region (amide I) revealed a differential pattern and percentage of specific secondary structures in LDL from patients with FH. Notably, we reported for the first time two distinct components at 1658 cm⁻^1^ and 1651 cm⁻^1^ in ApoB100 that were inversely altered in FH patients compared to control subjects. Interestingly, these double components have been previously identified in secretory phospholipase A2 (35) and in the transporter Melibiose Permease (46), both of which are proteins associated with lipid bilayers. Previous studies attributed the 1658 cm⁻^1^ component as more flexible and dynamic α-helices, whereas the 1651 cm⁻^1^ component was assigned to more stable α-helices. The increased presence of the 1651 cm⁻^1^ component in ApoB100 of hypercholesterolemic LDL suggests a reduced capacity for conformational restructuring in LDL from FH patients, potentially affecting their adaptability to phospholipolysis or other enzymatic modifications of LDL in the arterial intima.

The strong and highly significant correlation between CEs and α-helices in ApoB100 suggests that CE plays a major role in influencing the structural conformation of α-helices. Our findings align with a molecular LDL models previously proposed that show a physical interaction between radially oriented CE molecules in the outer layer of the LDL lipid core and the α-helical domains of ApoB100 (69, 70). Additional studies have highlighted the crucial role of CE in LDL remodeling, particularly those linking CE transfer protein, LDL remodeling, and risk factor profiles in patients with FH (71, 72). Our results support the notion that CE enrichment in LDL particles alters CE/ApoB100 interactions in the LDL of FH patients, leading to a replacement of flexible α-helices with more stable α-helical structures in ApoB100. This change could contribute to the increased propensity of LDL to aggregate in FH patients. The inverse relationship between LDL aggregation susceptibility, the CE content, and the flexible α-helices in ApoB100 holds significant translational implications for cardiovascular research. First, it is essential to investigate whether statins and PCSK9 inhibitors can enhance the proportion of flexible α-helices by reducing CE levels. Second, developing novel compounds that preserve the integrity of these flexible α-helices, even in the presence of elevated CE levels, could provide a promising strategy for mitigating cardiovascular risk in patients with FH. Importantly, this increased susceptibility to LDL aggregation, observed not only in adults but also in children with FH (73), underscores the urgent need for interventions that can address these molecular and structural challenges across all age groups.

An intriguing question remains whether all small dense LDL (sdLDL) particles undergo the same specific remodeling of α-helices in ApoB100. sdLDL particles are a key feature of the atherogenic lipoprotein phenotype, often linked to conditions such as insulin resistance, type 2 diabetes, and metabolic syndrome (74). These particles are particularly concerning due to their increased arterial penetration, enhanced retention in the intima, higher susceptibility to oxidation and aggregation, and diminished binding affinity for the LDL receptor. The predominance of sdLDL has been firmly established as an independent risk factor for CVD (75, 76), highlighting the need for further exploration into how structural changes in ApoB100 might influence their pathogenic role.

**Fig. 8:**
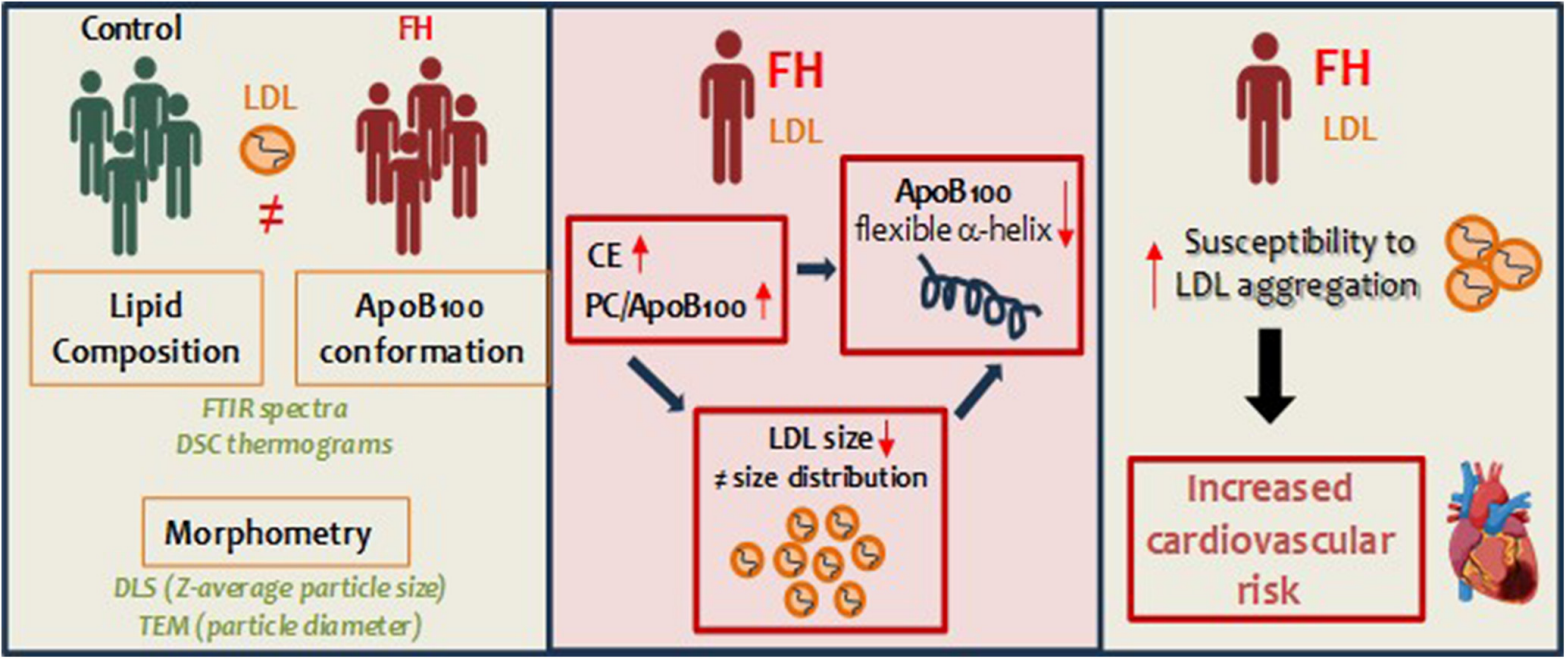
Summary of the main findings of the study. A more rigid and stabilized cholesteryl ester (CE) core in hypercholesterolemic LDL leads to reduced particle size and a loss of the flexible α-helix component in ApoB100. This structural alteration significantly increases LDL's susceptibility to aggregation in patients with familial hypercholesterolemia (FH), contributing to the heightened cardiovascular risk observed in this population. These findings underscore the critical role of LDL composition and conformation in its pathogenic behavior, offering new insights into potential therapeutic strategies targeting LDL stability and aggregation. CE, cholesteryl esters, FH, familial hypercholesterolemia.

In conclusion, our study demonstrates that the increased susceptibility of LDL to aggregation in patients with FH is closely linked to a heightened and stabilized CE core that reduces LDL size and promotes a loss of a specific flexible α-helix component in ApoB100 (summarized in Fig. 8). Our findings help elucidate the elevated risk of ASCVD in FH patients and suggest that ApoB100 remodeling could be a valuable therapeutic target not only for FH but also for a wide range of metabolic disorders.

### Data availability

Data will be shared upon reasonable request (Vicenta Llorente Cortés, Vicenta.llorente@iibb.csic.es; cllorente@santpau.cat).

## Supplementary material

Supplementary material is available online (supplemental Figs. S1-S3).

## Conflict of interest

The authors declare that they have no conflicts of interet with the contents of this article.
