# Supplementary material for: ApoB100 remodeling and stiffened cholesteryl ester core raise LDL aggregation in familial hypercholesterolemia patients
Source: J Lipid Res. 2024 Nov 16;66(1):100703. doi: 10.1016/j.jlr.2024.100703 (PMC11731490; doi:10.1016/j.jlr.2024.100703)
Supplement: Supplementary file 1 [file mmc2.docx]

Figure S1

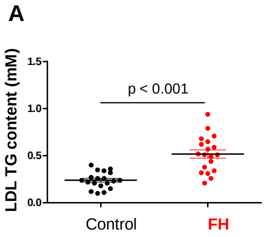

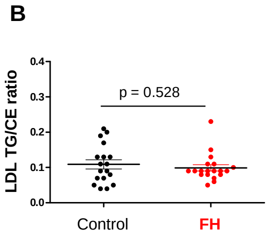

Figure S1. Graphs showing the comparison of TG content and TG/CE ratio in LDL from patients with FH and controls. Data are shown as mean ± SD. For comparison of two groups, the unpaired 2-tailed Student's t-test (for unequal variances) or two-way ANOVA followed by Tukey's post hoc test (for normality and variance homogeneity) was used. Differences were considered statistically significant when $p < 0.05$. CE, cholesteryl esters; TG, triglyceride; FH, familial hypercholesterolemia.

Figure S2

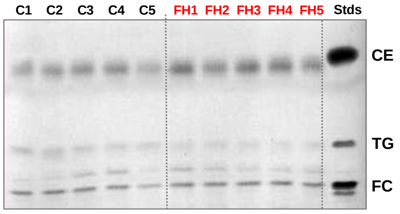

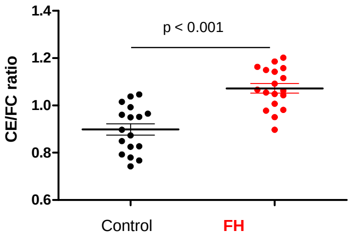

Figure S2. Representative thin-layer chromatography (TLC) showing the CE, TG, and FC content in LDL from control (C1-C5) and FH patients (FH1-FH5) with graphs showing the differences in the CE/FC ratio in LDL from each group. Data are shown as mean SD. For comparison of two groups, the unpaired 2-tailed Student's t-test (for unequal variances) or two-way ANOVA followed by Tukey's post hoc test (for normality and variance homogeneity) was used. Differences were considered statistically significant when $p < 0.05$. Stds, standards; CE, cholesteryl esters; TG, triglycerides; FC, free cholesterol; FH, familial hypercholesterolemia; FTIR, Fourier-transform infrared spectroscopy.

Figure S3

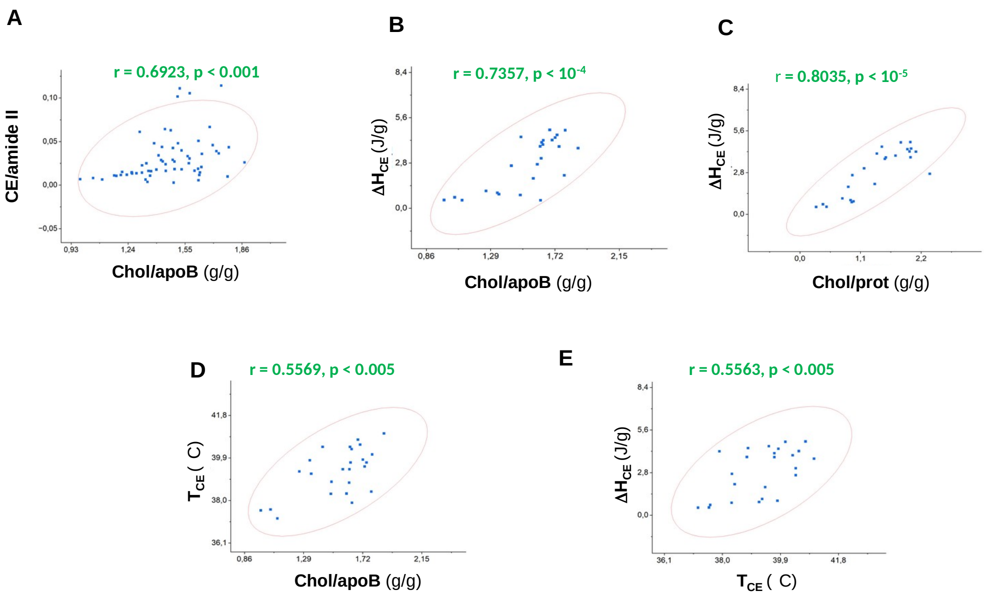

Figure S3: Graphs demonstrating the high correlation between biophysical and biochemical lipid LDL parameters. The graphs show the correlation between: (A) the ratio of FTIR band areas (A800 cm^{-1}/A1700 cm^{-1}) assigned to CE/protein and the ratio of cholesterol/apoB100 biochemically determined, (B) the normalized enthalpy of the smectic-to-disorder phase transition associated with the area of the lipid CE core peak (ΔH_{CE}) and the ratio of cholesterol/apoB100 biochemically determined, (C) the normalized enthalpy of the smectic-to-disorder phase transition associated with the area of the lipid CE core peak (ΔH_{CE}) and the ratio of cholesterol/proteins biochemically determined, (D) the transition temperature of the CE core (T_{CE}) and the ratio of cholesterol/apoB100 biochemically determined, (E) the transition temperature of the CE core (T_{CE}) and the normalized enthalpy of the smectic-to-disorder phase transition associated with the area of the lipid CE core peak (ΔH_{CE}). Spearman correlation coefficients (r) and statistical significance (p) are shown. FH, familial hypercholesterolemia.

Figure S4

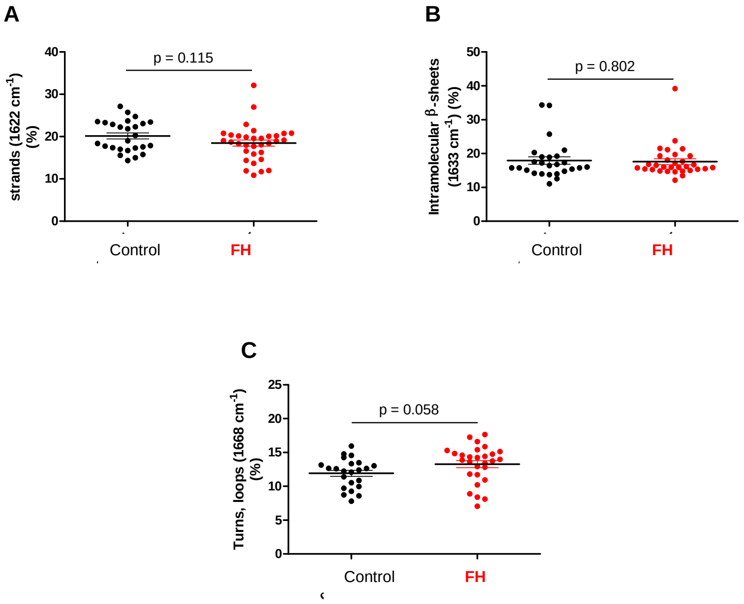

Figure S4: Graphs comparing the percentage of specific ApoB100 secondary structures in LDL from patients with FH and control subjects. The graphs display differences in the percentage of β-strands (A1622 cm^{-1}) (A), intramolecular β-sheets (A1633 cm^{-1}) (B), and turns/loops (1688 cm^{-1}) (C). For the comparison between control and FH groups, a two-way ANOVA followed by Tukey's post hoc test was used. Results are presented as mean SD. Differences were considered statistically significant when $p < 0.05$. FH, familial hypercholesterolemia.

Figure S5

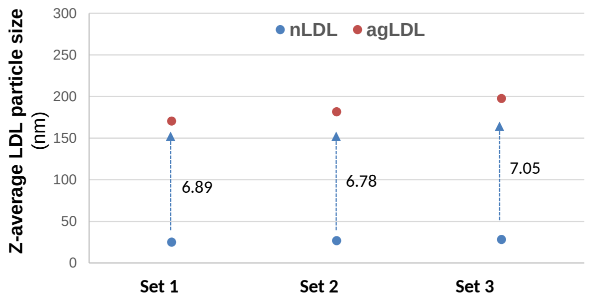

Figure S5. Plots showing the variation in Z-average LDL particle size for unmodified LDL (nLDL) and LDL modified by sphingomyelinase (SMase) treatment (agLDL). LDL was isolated from a human normocholesterolemic pool and used as an endogenous control in three different sets of human sample measurements. The values in the middle of the arrows indicate the fold change in Z-average particle size caused by SMase treatment. Each data point represents the mean of duplicate measurements. **nLDL** refers to unmodified LDL, while **agLDL** refers to LDL aggregated by SMase. The intra-assay coefficient of variation (CV) for the Z-average size of nLDL, agLDL, and the fold change was less than 15%, demonstrating the precision of the measurements.
